# Editorial: Rising stars in tuberculosis

**DOI:** 10.3389/ftubr.2025.1635563

**Published:** 2025-07-01

**Authors:** Harald G. Wiker, Elsa Anes, Philippe Clevenbergh, Seyed Ehtesham Hasnain, Sven Hoffner, Alissa C. Rothchild, Vishwanath Venketaraman

**Affiliations:** ^1^Faculty of Medicine, University of Bergen, Bergen, Norway; ^2^Research Institute for Medicines, Faculty of Pharmacy, Universidade de Lisboa, Lisbon, Portugal; ^3^Infectious Diseases Department, CHU Brugmann, Brussels, Belgium; ^4^Department of Biochemical Engineering and Biotechnology, Indian Institute of Technology Delhi, New Delhi, India; ^5^Department of Global Public Health, Karolinska Institutet, Solna, Sweden; ^6^Department of Veterinary and Animal Sciences, University of Massachusetts Amherst, Amherst, MA, United States; ^7^Department of Basic Medical Sciences, Western University of Health Sciences, Pomona, CA, United States

**Keywords:** *Mycobacterium tuberculosis*, tuberculosis, multi-drug resistance (MDR), glutamine metabolism, antigenic variation, mucosal immmunity, bacterial fitness, transcriptional regulatory network (TRN)

## Introduction

Tuberculosis (TB) remains a formidable global health challenge, particularly with the emergence of multidrug-resistant TB (MDR-TB) and even more resistant forms of TB. New and innovative approaches are needed to fight this epidemic. This editorial showcases a diverse selection of research papers published by some of the future leaders in the field of tuberculosis and mycobacterial diseases, highlighting advancements in understanding and combating TB. From genetic patterns of MDR-TB in Russia to the role of glutamine metabolism in TB pathogenesis, these studies offer valuable insights and new perspectives from TB researchers in the early stages of their careers. We explore the unique functionalities of *Mycobacterium tuberculosis*' PE/PPE proteins, the potential of mucosal BCG vaccination, and innovative machine learning models predicting TB fitness. Together, these contributions underscore the multifaceted efforts and breakthroughs in TB research, aiming to improve diagnosis, treatment, and 16 prevention strategies. This Research Topic highlights the rising stars in the field who are leading the way towards novel solutions for an ancient and deadly disease.

## Multi-drug resistant tuberculosis

MDR-TB is a significant global health challenge due to its complex treatment, high cost, and severe health outcomes. Understanding the mechanisms of drug resistance and addressing the factors contributing to its development are crucial for controlling the spread of MDR-TB. In Sverdlovsk Oblast in Russia, MDR-TB is a considerable problem. Genetic patterns were used to study transmission dynamics of MDR-TB cases by Umpeleva et al.. The study found that a significant proportion of *M. tuberculosis* isolates had unique genetic patterns, suggesting minimal recent transmission. The presence of mutations in gyrA/B genes, associated with fluoroquinolone resistance, was more common in isolates with unique patterns, indicating that these mutations may have developed in already drug-resistant strains due to inadequate chemotherapy regimens but had not yet circulated widely, which could be due to biological fitness costs related to these mutations. Investigating the gyrA/B mutations may provide insights into how *M. tuberculosis* adapts to drug resistance and maintains its transmissibility.

*M. tuberculosis* infection can be transmitted to the fetus via hematogenous spread from the placenta to the umbilical vein or through the aspiration or ingestion of infected amniotic fluid. Congenital TB is a relatively rare condition compared to other forms of TB but carries a high mortality rate. The incidence of congenital TB is higher in regions with high TB prevalence, such as parts of Asia and Africa. This case report by Zhang et al. of a neonate girl with congenital MDR-TB, describes the challenges associated with managing this rare condition. The infant's mother, who underwent *in vitro* fertilization–embryo transfer (IVF-ET), was later found to have MDR-TB affecting both her lungs and reproductive system. The report underscores the importance of early diagnosis and prompt treatment to improve outcomes for both the mother and the infant.

## The role of glutamine metabolism in the pathogenesis of tuberculosis

Glutamine is the most abundant amino acid in the human body and is found in high concentrations in the blood and tissues. Glutamine metabolism plays a critical role in the pathogenesis of *M. tuberculosis* as delinated by Parveen and Bishai. *M. tuberculosis* manipulates host metabolic pathways, including glutamine metabolism, to establish infection, progress, and disseminate within the host. Recent findings demonstrate significant alterations in glutamine metabolism in TB patients and infected macrophages, and this underscores the importance of glutamine as a nitrogen donor and its role in immune cell function during *M. tuberculosis* infection. Inhibition of glutamine metabolism can enhance the antimycobacterial activity of macrophages and improve lung histopathology and survival in murine models of TB. Consequently, there is a potential for targeting glutamine metabolism as a novel host-directed therapy for TB. Also, lower circulatory glutamine levels are identified as a potential diagnostic marker for distinguishing active TB patients from latent TB patients and healthy controls.

## “Personal protective equipment” of *M. tuberculosis*

The PE (Pro-Glu) and PPE (Pro-Pro-Glu) proteins are highly variable and polymorphic, contributing to antigenic variation. They are almost exclusively found in the genus *Mycobacterium* and a few other *Actinomycetales*, such as *Rhodococcus* and *Nocardia*, but in far fewer numbers. No equivalent families are found in Gram-negative bacteria or typical commensal microbiota, making them a unique hallmark of the mycobacterial genome. The large number (~10% of the *M. tuberculosis* genome) and high sequence similarity make PE/PPE proteins difficult to study experimentally. The paper by Resstel et al. highlights several of the unique functionalities of PE and PPE proteins. They can interfere with host immune signaling and help the bacteria persist in the host over long periods. This allows mycobacteria to evade the host defense mechanisms by altering surface-exposed antigens. The paper provides detailed insights into several well-characterized PE/PPE proteins, such as PPE2, PE_PGRS33, PE_PGRS47, PE_PGRS29, and MirA. These proteins have been shown to affect various host cell pathways, including reactive oxygen species (ROS) production, nitric oxide (NO) production, autophagy, and actin tail formation.

## Mucosal BCG vaccination

When BCG was first developed by Albert Calmette and Camille Guérin in the early 1900s, it was initially administered orally. The first human dose of BCG was given orally to an infant on July 18, 1921. However, the efficacy of oral BCG varied, and it was eventually replaced by intradermal administration, which became the standard practice. Studies have demonstrated that mucosal BCG vaccination can induce lung-resident memory T cells and provide significant protection against *M. tuberculosis* infection in animal models. This has led to renewed interest in exploring mucosal delivery as a potential strategy for enhancing vaccine efficacy as advocated by Larsen et al.. The study demonstrates that intranasal delivery of the BCG vaccine provides significant protection against pulmonary TB and enhances survival across various mouse strains with different susceptibilities to TB. It is important to further explore the specific immune mechanisms and correlates of protection induced by mucosal BCG vaccination as compared to those induced by traditional intradermal vaccination.

## Predicting fitness in *M. tuberculosis*

Bustad et al. assembled a large and biologically diverse RNA expression compendium, integrating data from various sources to infer a comprehensive transcriptional regulatory network (TRN) for *M. tuberculosis*. This network includes 214 transcription factors and 3,978 genes, representing a significant advancement over previous models. The study introduces a novel machine learning model that predicts *M. tuberculosis* fitness based on TFA profiles derived from gene expression data. This model can accurately predict growth arrest and resumption under hypoxia and reaeration conditions. The model elucidates the transcriptional programs driving *M. tuberculosis* growth phenotypes, identifying key transcription factors involved in stress adaptation. This provides a deeper understanding of the molecular mechanisms underlying *M. tuberculosis*' response to environmental changes.

Transcriptional regulatory network informed interpretable machine learning can also be a powerful approach to study the fitness of *M. tuberculosis* strains with antibiotic resistance mutations and is certainly a tool that can bring further insight into the development and transmission of drug resistance in TB.

